# Automated Genotyping of Biobank Samples by Multiplex Amplification of Insertion/Deletion Polymorphisms

**DOI:** 10.1371/journal.pone.0052750

**Published:** 2012-12-27

**Authors:** Lucy Mathot, Elin Falk-Sörqvist, Lotte Moens, Marie Allen, Tobias Sjöblom, Mats Nilsson

**Affiliations:** 1 Department of Immunology, Genetics and Pathology, Science for Life Laboratory, Uppsala University, Uppsala, Sweden; 2 Department of Biochemistry and Biophysics, Science for Life Laboratory, Stockholm University, Solna, Sweden; University of Sydney, United States of America

## Abstract

The genomic revolution in oncology will entail mutational analyses of vast numbers of patient-matched tumor and normal tissue samples. This has meant an increased risk of patient sample mix up due to manual handling. Therefore, scalable genotyping and sample identification procedures are essential to pathology biobanks. We have developed an efficient alternative to traditional genotyping methods suited for automated analysis. By targeting 53 prevalent deletions and insertions found in human populations with fluorescent multiplex ligation dependent genome amplification, followed by separation in a capillary sequencer, a peak spectrum is obtained that can be automatically analyzed. 24 tumor-normal patient samples were successfully matched using this method. The potential use of the developed assay for forensic applications is discussed.

## Introduction

Large biobanking efforts, in particular in cancer research, have presented a new genotyping challenge and a need for a technique to simply and quickly verify that paired samples are from the same patient before any further analyses are undertaken. In cancer research not only is it desirable to correctly match samples from the same patient, but also to provide some information on the genomic stability of the tumor sample already at an early stage of analysis.

The analysis of simple tandem repeat polymorphisms (STRs) became the genotyping method of choice in the 1990s. STRs are di-, tri- or tetranucleotide repeat sequences showing high levels of allelic variation in the number of repeat units. They are polymorphic markers that are widely and evenly distributed across the human genome and can be typed using PCR amplification. This trend changed towards the end of the 1990s with the increase in the use of single nucleotide polymorphisms. SNPs are highly abundant and are more stable than STRs due to lower mutation rates. They are, however, biallelic and therefore less informative than STRs.

Small insertion and deletion (indel) polymorphisms have recently been of particular interest for genotyping as they combine the desirable features of both SNPs and STRs. They are well conserved with low mutation rates, widely distributed throughout the genome, suitable for high throughput analyses (even in degraded samples) and are polymorphic within and between populations [1]. They also may be studied using simple PCR based methods, unlike conventional methods used to study SNPs [1]. The presence or absence of a certain number of targeted deletions and insertions with a population prevalence of between 0.3 and 0.7 can also be utilized as a reliable technique for ascertaining identity or confirming matching samples from the same patient, while minimizing the amount of genetic information revealed [2]. However, as they are less informative than multiallelic markers, indels are rarely used in commercial genotyping techniques. In fact, 3–5 fold more indels than STR markers have to be analyzed in order to obtain the same power of discrimination which will require more template DNA [3].

In this paper we describe the development of a robust multiplex technique for detection of insertion/deletion polymorphisms. Multiplex ligation-dependent genome amplification (MLGA) is a targeted approach based on a technique originally described by Dahl *et al* and developed by Isaksson *et al* [4], [5]. The procedure is based on the hybridization of oligonucleotide constructs, called selector probes, to defined target nucleic acid sequences. The selectors contain target-complementary end-sequences, joined by a linking sequence (vector), and they act as ligation templates to direct circularization of target DNA fragments containing indels. The circularized targets are then amplified in multiplex using universal PCR primer pairs specific for the general linking sequence in the selectors [4]. Compared to traditional methods, this technique offers the advantage of facile probe production. The probe length is 75–90 nucleotides and requires no modifications or purification. Also, only one probe is required per target locus, imparting a kinetic advantage as successful hybridization of one end automatically holds the other end close to its respective target. This proximity effect increases the speed of the hybridization reaction, thereby decreasing reaction times [5].

Cancer is a genetic disease with an unstable genome. This is as a result of an acquirement of mutations and alterations in genes regulating growth and proliferation. Genomic instability in cancer may be divided into two categories, chromosomal instability (CIN) and microsatellite instability (MSI). Chromosomal instability is complex; it affects widespread regions of the genome and is implicated in most solid tumors [6]–[8]. An average colorectal, breast, pancreatic or prostate cancer may lose 25% of their alleles [9]. In CIN positive tumors, it is not unusual for 75% of alleles to be lost [9]. In colorectal cancer (CrC) for example, 80–85% of cancers are CIN and exhibit a loss of heterozygosity upon comparison of affected regions from tumor and normal material [10]. Loss of heterozygosity can be useful to study in cancer, in particular for use in differentiating between CIN and MSI in CrC, predicting prognosis and what treatments are most suitable. The MLGA technique described in this paper aims to provide information both on concordance between samples and LOH (loss of heterozygosity) analysis for tumor samples.

## Materials and Methods

### Ethics Statement

The study was approved by the Regional Ethical Review Board of Uppsala (2007/116 and 2009/224), written consent was obtained from participants and patient data was analyzed anonymously.

### DNA samples

DNA was extracted from 48 tumor and normal fresh frozen colon tissue samples on a Tecan Evo MCA 150 robotic platform using the extraction method described in Mathot *et al* (2011) [11]. Colorectal tissue samples were obtained from the frozen tissue collection at the Department of Pathology, Academic Hospital Uppsala. Commercial genomic DNA (a pooled sample from male donors) from ProMega (Article No. G1471) was also used as a control DNA in this study. In addition, DNA from an FFPE (formalin fixed, paraffin embedded) tissue sample was extracted using a QIAamp DNA FFPE Tissue Kit (Qiagen) according to manufacturer's instructions.

### Target selection

All human genetic variations reported in dbSNP (GRCh37, http://www.ncbi.nlm.nih.gov/projects/SNP/) were downloaded from the NCBI ftp-site on 20^th^ July 2011. Out of all genetic variations the non-homopolymeric 3 to 5 base pair insertions and deletions with a prevalence of 30–70% in a European population were retrieved, giving a pool of 500 possible insertions and deletions to choose from. Using in-house developed software based on the operating principles of PieceMaker and Disperse, a set consisting of 70 insertions and deletions was selected from the pool [12], [13]. Each insertion and deletion was located in a Dde I/Hin1 II restriction fragment. The restriction fragments were 100–300 bp long with at least one fragment on each of 21 autosomes. All insertions and deletions included in the design were from the same European population (Marshfield, population ID 484). For sex determination, we included a target on each of the amelogenin genes, *AMELX* and *AMELY*, each producing a different length fragment, 109 and 106 bps respectively (17). A summary of the targeted deletions and insertions is shown in Table S1. The selected fragments were divided into three panels such that a ladder with peak distances of 6–23 bp would be obtained upon multiplex amplification (18). Two panels targeted deletions, while the remaining panel targeted insertions. The population data for the selected insertion/deletion markers is shown in Table 1, where a combination of all 3 panels gives a cumulative power of discrimination as calculated for forensic analysis.

**Table 1:** Population data for insertion/deletion markers.

	Panel 1	Panel 2	Panel 3	Panels 1, 2 and 3
**Average match probability ** [**20**]	4.60×10^−8^	2.11×10^−7^	1.85×10^−8^	1.80×10^−22^
**Average expected heterozygosity ** [**21**]	0.47	0.49	0.47	0.48
**Power of discrimination (1- MP)**	99.9999954%	99.9999789%	99.9999981%	>99.999999999%
**Power of exclusion (trio) ** [**22**]	97.19%	96.13%	97.69%	99.997%
**Average paternity index ** [**20**]	26.18	23.02	31.92	19237.05

Panels 1, 2 and 3 consist of approximately 18 target indels each, and are outlined in detail in Table S1.

### MLGA probe design

**Figure 1:**
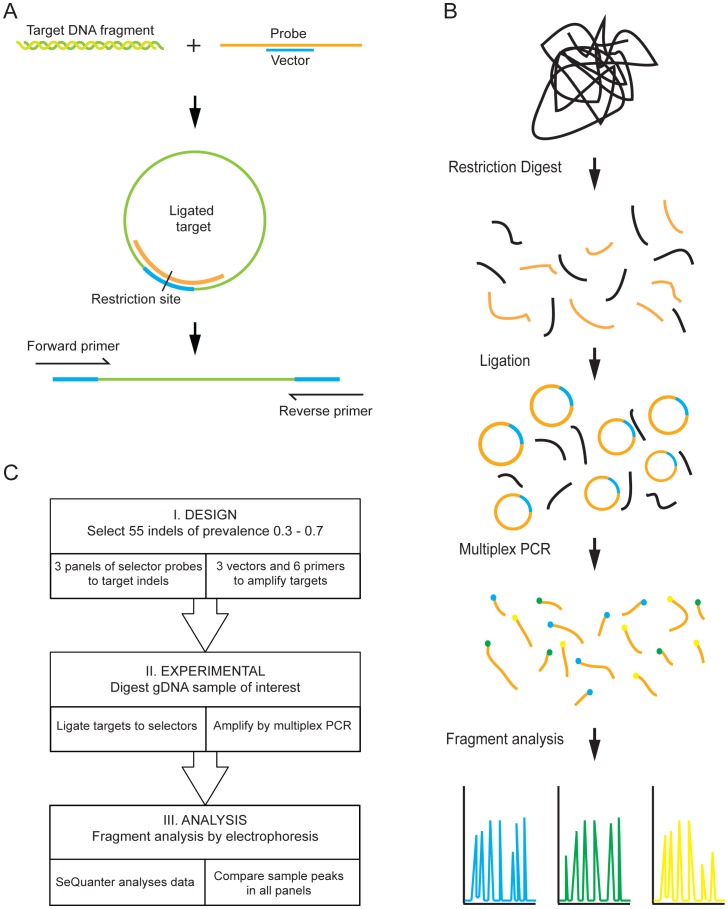
An overview of the experimental design and procedure. **A** shows the role of the selector probe and complementary vector. The target DNA fragment containing the insertion/deletion is cut with restriction enzymes and ligated to a complementary probe to form a circle. The circular ligation product is again cut to form a linear fragment with universal primer binding site. **B** shows the MLGA reaction scheme. Genomic DNA is restriction digested; ligated to specific selector probes and these products are amplified by multiplex PCR using fluorescent labels. The fragments can then be separated by capillary electrophoresis and analyzed. **C** is a schematic representation of the process, from design to analysis.

MLGA probes for target fragment circularization were designed using ProbeMaker software (19). Each MLGA probe was ∼90 nucleotides long, consisting of two target specific arms with a panel specific sequence in between (Table S2). The complementarity between the target specific arms and the arms of a selected restriction fragment made selection and circularization of the restricted gDNA possible. Upon hybridization of the panel specific sequence to its complementary vector a recognition site for the restriction enzyme Hind III and primer sites for the multiplex PCR amplification were formed (Figure 1A).

Three universal primer sequences were used for fragment amplification by PCR (Table 2). The universal primers were designed using a non-human DNA template (*Escherichia coli* str. K12 substr. DH10B) and tested for equal amplification efficiency using this template, whilst ensuring that there was no amplification of interfering size using human gDNA template (Promega) (Figure S1). The forward primers were then conjugated to each of one of the 3 fluorophores, FAM, NED, or VIC (Sigma-Aldrich, Applied Biosystems).

**Table 2:** Sequences for universal primers, vectors and between probe arms.

Universal Primer	Sequence 5′-3′	Corresponding vector sequence 5′-3′	Between selector probe arms 5′-3′
**Vector1_fwd_FAM**	agcttcggccgatgccaaat	atttggcatcggccgaagcttcgatcgcatcgagca	tgctcgatgcgatcgaagcttcggccgatgccaaat
**Vector1_rev**	gtttcttagcttcgatcgcatcgagca		
**Vector2fwd_NED**	agcttaaaccgcaccgttgg	ccaacggtgcggtttaagcttcgacgcggatttcgt	acgaaatccgcgtcgaagcttaaaccgcaccgttgg
**Vector2_rev**	gtttcttagcttcgacgcggatttcgt		
**Vector3_fwd_VIC**	agctttcggcgacggtttca	tgaaaccgtcgccgaaagctttgcgtcgtcgcatca	tgatgcgacgacgcaaagctttcggcgacggtttca
**Vector3_rev**	gtttcttagctttgcgtcgtcgcatca		

### Multiplex ligation dependent genome amplification

Genomic DNA samples were first fragmented using a restriction digestion at 37°C for 1 hour using 2 U of restriction enzymes Dde I and Hin1 II in a 10 µl reaction mixture containing 1× Buffer Tango (Thermo-Scientific). The enzymes were subsequently inactivated at 80°C for 20 min. Circularization and ligation of restriction digested fragments was performed in a 20 µl reaction by adding 2.2 nM vector oligonucleotide, 0.1 nM of each Selector probe, 9.67 mM MgCl_2_, 0.8 mM NAD, 4 U Ampligase (Epicentre) and 1× *Taq* DNA Polymerase PCR Buffer (Invitrogen) to the DNA. The reaction was incubated at 95°C for 5 minutes, followed by 90 min at 60°C. Amplification of these circularized target fragments was performed by adding 4 µl of the ligation product (∼40 ng DNA) to 21 µl of a PCR reagent mixture consisting of 0.25 mM dNTPs, 2.5× PCR buffer (Invitrogen), 0.5 mM MgCl_2,_ 0.5 µM each of forward and reverse primers, 5 U of Hind III (Thermo-Scientific) and 1.5 U Platinum *Taq* DNA Polymerase (Invitrogen). Cycling parameters were 37°C for 30 min, 5 min 95°C followed by 30–40 cycles of 95°C for 30 s, 60°C for 30 s, 72°C for 1 min followed by 10 min at 72°C. The cycling was performed on an Applied Biosystems 2720 Thermal Cycler.

Fluorescently labeled PCR products were analyzed by fragment analysis in a capillary sequencing instrument (ABI PRISM 3730xl) using LIZ500 (Applied Biosystems) as size standard followed by peak identification using the in-house developed SeQuanter software (Falk-Sörqvist *et al*, manuscript in preparation). The peak heights obtained were compared between the samples to confirm that individuals can be typed on the basis of these targeted deletions. This was done by digitalizing the peak output data and comparing paired samples to ensure a high level of concordance (i.e. a measure of how similar two DNA samples are to one another) regarding presence/absence of target amplicons.

For peak digitalization, a peak was reported as one (present) if the background peak height was less than a third of the amplicon peak height and the amplicon peak height was at least 0.1 of the mean amplicon peak height for the sample panel. If a peak was absent based on the above criteria it was reported as zero. Concordance between samples was then calculated from the digitalized peaks and only taking markers which had at least one peak present in both of the compared samples into consideration. This was to ensure only amplified markers were included in the comparison. A peak was counted as concordant if it was reported as present or absent in both samples. If a peak was present in one sample and absent in the other it was considered discordant. The concordance of a sample pair was then reported as the fraction of concordant peaks.

## Results

The MLGA technique presented here aims to establish and validate a high throughput genotyping method primarily for fast, parallel analysis of DNA extracted from biobanked tissue samples. The experimental procedure is outlined in Figure 1B and consists of four main steps; *(1)* restriction digestion of genomic DNA, *(2)* ligation and circularization of selectors to target fragments, *(3)* multiplex amplification by PCR and *(4)* fragment analysis by capillary electrophoresis. By using a multiplex ligation dependent amplification approach as described by Isaksson et al, the amount of template DNA can be reduced compared to running a large number of simplex reactions [5], [14]. The entire process, from design to analysis, is briefly outlined in Figure 1C. Probes specific for the target indels were initially evaluated in simplex reactions in order to test that each one could successfully amplify the correct region and produce a PCR product of the correct length. The individual amplicons from simplex reactions are shown in Figure S2.

To demonstrate the sensitivity of the assay, a number of serial dilutions of gDNA from the same DNA sample (ProMega) were tested, with input DNA ranging from 40 ng to 0.3125 ng. The assay showed reproducibility with input of 0.3125 ng DNA, i.e. the fragment profile was maintained at this level of DNA input when compared with the standard method. The peak profiles are shown in Figure S3. There was an allelic dropout of 6.5% from 40 ng input to 0.3125 ng input and the fluorescence units absorbed by the highest peak (150 bps) decreased by 25%.

The MLGA method was evaluated by performing a restriction digest on tumor and normal matched genomic DNA from 24 individuals with colorectal cancer from a Swedish population. The inclusions of an amelogenin gene target on both X and Y chromosomes allowed us to also identify the gender of each individual, as the *AMELY* target was only amplified in males. The SeQuanter program correctly matched 24/24 genders and the results are shown in Table 3.

**Table 3:** Concordance of 24 tumor/normal matched pairs.

Tumor/Normal Pair	Concordance (%)	Actual Match Probability	Number of peaks compared	Number of peaks showing possible LOH	Gender
**31/32**	90.2	5.1×10^−24^/5.8×10^−24^	92	4	F
**57/58**	97.8	3×10^−22^/1.9×10^−24^	90	0	M
**75/76**	96.8	6.4×10^−19^/9.7×10^−20^	94	1	M
**87/88**	97.8	1.6×10^−22^/4.5×10^−23^	92	1	F
**121/122**	93.8	2.3×10^−25^/8.6×10^−25^	96	4	F
**151/152**	94.7	3.6×10^−24^/1.1×10^−22^	94	2	F
**35/36**	92.6	1.6×10^−24^/3.6×10^−25^	94	3	F
**61/62**	93.9	1.3×10^−23^/3.1×10^−22^	98	4	F
**77/78**	89.6	2.6×10^−23^/4×10^−24^	96	0	M
**89/90**	96.7	6.8×10^−22^/5.4×10^−22^	90	2	M
**123/124**	94.7	1.7×10^−23^/1.3×10^−22^	94	5	M
**153/154**	92.7	1.7×10^−22^/2.9×10^−24^	96	3	M
**37/38**	94.7	7.9×10^−25^/1.3×10^−24^	94	1	M
**63/64**	96.8	6×10^−22^/4.8×10^−22^	94	2	F
**79/80**	94.6	1.1×10^−21^/6.6×10^−22^	92	1	F
**101/102**	95.0	5×10^−24^/1.6×10^−24^	100	4	M
**139/140**	99.0	1.7×10^−22^/2.3×10^−23^	96	0	M
**177/178**	97.0	1.5×10^−25^/9.4×10^−24^	100	3	M
**55/56**	94.7	6.6×10^−24^/1.3×10^−22^	94	4	M
**71/72**	85.6	6.5×10^−19^/9.1×10^−22^	90	5	M
**81/82**	87.2	5.6×10^−22^/1.4×10^−24^	94	6	M
**109/110**	94.6	1.6×10^−21^/7.3×10^−21^	92	4	F
**147/148**	89.8	5.9×10^−23^/8.3×10^−24^	98	8	M
**181/182** *	81.0	1.3×10^−21^/1.8×10^−24^	88	9	F

* Poor amplification for these samples.

A concordance of greater than 95% was seen when DNA from the same normal tissue was analyzed twice, confirming that the method can successfully match individuals (Table S3). The concordance between the 24 paired tumor/normal samples is shown in Table 3. For our purposes, T/N paired samples with a concordance of above 85% were considered correctly matched. This would be expected to be greater than or equal to 95% using DNA from normal cells, as shown, but tumor DNA is prone to loss of heterozygosity, resulting in a lower overall concordance. Unmatched tumor normal pairs were between 51 and 81% concordant (Table S4), and unmatched normal pairs were between 63 and 81% concordant (data not shown). Sample pair 181/182 (T/N respectively) showed a lower than expected concordance for a matching pair but manual peak analysis showed an overall poor amplification for these samples, with the result that fewer common targets were compared in the analysis (88 out of 108). However, comparing both 181 and 182 with all other samples did not produce a higher concordance with any other DNA profile. All samples have a higher concordance with their matched pair than with any other sample (Table S4).

The assay was also tested as described above using gDNA extracted from FFPE to assess the performance of the method using fragmented DNA. The method proved to be suitable for use even when the input sample is fragmented (sample concordance of 95% with two FFPE normal DNA samples of the same origin). There was however, a requirement for a higher input of FFPE DNA (>10 ng in the PCR reaction for best results). Decreasing sample input from 40 ng to 10 ng resulted in a 22% decrease in markers amplified and decreasing to 2.5 ng resulted in a 56% decrease in amplified targets. For lower template input amounts, there was a notable decrease in targets under 200 bps which provides an incentive for excluding probes targeting larger size products when using degraded DNA.

## Discussion

We have developed a method for genotyping that is non-labor-intensive using the selector-based technique, multiplex ligation dependent genome amplification. The MLGA technique involves the amplification of targeted fragments of digested genomic DNA using oligonucleotide probe molecules and has previously proven to be a suitable method for the analysis of CNVs [5], [15], [16]. We describe a further development of the procedure and demonstrate that the method is a suitable tool for genotyping by targeting selected indels. The development of the MLGA technique described here, allows for more targets to be included in one multiplex reaction by using three vector molecules instead of one [5]. 48 samples were run simultaneously, illustrating the scalability of the technique. The method is all carried out in one reaction vessel and thus could be implemented on a robotic platform capable of pipetting the various reagents in a 96 well format.

The technique can be used for input DNA amounts of less than 0.4 ng, illustrating a possible application for identification in forensic samples where there may be a limited amount of input genomic material. However, the use of this method still needs to be evaluated for forensic use. The procedure is also efficient, with the entire assay taking less than 5 hours in total to perform, with minimal hands on time.

Targeting indels rather than microsatellites in cancer specimens results in more reliable and reproducible results due to their stability and lower mutation rates [17]. Targeting indels is also more appropriate when dealing with degraded samples compared to STRs, as shorter fragments may be amplified [18], [19]. There have recently been developments in other technologies that are also targeting insertion and deletion polymorphisms for genotyping purposes, e.g. the PCR based Investigator DIPplex Kit from Qiagen. The MLGA method developed in this paper has a larger number of markers resulting in a match probability of 1.80×10^−22^ which has greater discrimination power than the markers used in the DIPplex kit (match probability of 3.3×10^−13^) [18]. We have also focused on targeting short indels (between 3 and 5 nucleotides) in order to reduce allelic drop out, which is of particular importance in degraded samples. The DIPplex kit includes indels of up to 22 base pairs. The number of deletions and insertions targeted by multiplexing here result in a genotyping tool comparable to routine forensic STR analysis and one sensitive enough even for forensic analysis, due to the small quantities of template DNA needed and reduced frequency of allelic dropout in degraded samples by targeting short indels. It is also possible to analyze FFPE samples with this method, which is useful for archived material, in particular if one reduces the indels targeted to those producing products of less than 200 bps. This would result in a test of 15 markers with a power of discrimination greater than 99.9999%.

The peak profiles of the same individual show the same pattern for the targeted indels, as expected, and also demonstrate the ability of the method to detect concordance between paired samples. Lower levels of concordance may sometimes be explained by a loss of heterozygosity in the tumor sample. One can distinguish between a low concordance as a result of a real mismatch, a highly instable tumor with a high level of LOH or simply a poor amplification in a number of ways. A true mismatch should show a higher level of concordance between the sample in question and another sample that is not supposed to be the matching one. Tumor samples with a high LOH should not match another sample with a higher level of concordance than the true match (even if the concordance of the true match is lower than expected), and this can been seen when comparing concordances between all samples. A poor amplification will be evident from the peak profile. However, even if a reaction results in a poor amplification, each correct pair should still be possible to match by comparing to all other samples in the data set, as we have shown for samples 181/182 (Table S4).

It is important to acknowledge that if this method is set up manually, the experimental work is comparable to that of using an STR profiling kit. The MLGA method however greatly simplifies the analysis of the output, reducing the series of peaks obtained to one concordance value, without using expensive software. Compared to other indel genotyping methods, advantages of the present MLGA based technique include *(1)* ability to target a large amount of targeted insertions and deletions in a single-vessel reaction, *(2)* large number of markers to increase discrimination for forensic use *(3)* automated data analysis due to the simplicity of peak detection that does not require expensive software (the SeQuanter program used will be open access), *(4)* possible automation of sample processing and *(5)*, a low match probability of 1.80×10^−22^ for all markers combined, giving a reliable power of discrimination.

## Supporting Information

Figure S1 **Universal primers designed against** ***E.Coli*** **genome do not amplify products of the same size in human gDNA.** PCR products amplified using the universal primer sequences were run on a 1% agarose gel and stained with SYBRsafe. Positive control was human gDNA with amplification of *PRPS1* exon 4. DH10B *E.Coli* DNA was used as template for the multiplex amplification of 3 primer pairs to ensure equal efficiency of primers. Human gDNA was used as template to check for unwanted PCR products of the same size as target fragments.
(TIF)

Figure S2 **Each fragment containing a targeted insertion/deletion successfully amplified in simplex.** 55 targets were amplified by a simplex MLGA reaction to ensure all could produce a PCR product before the probes were pooled. Each product was run on a 1% agarose gel. **A, B** and **C** show simplex products from panels 1, 2 and 3, respectively.
(TIF)

Figure S3 **Peak profiles from SeQuanter show that the MLGA method is robust with input DNA of less than 1 ng.** **A, B C** and **D** are profiles of amplified targets using input of 40, 10. 2.5 and 0,625 ng of gDNA, respectively.
(TIF)

Table S1 **Targeted deletions and insertions.** Chromosome position according to dbSNP GRCh37, July 2011.
(DOCX)

Table S2 **Probe sequences.**
(DOCX)

Table S3 **Concordances of above 95% using DNA from the same non-tumor tissue.**
(DOCX)

Table S4 **Percentage concordances of all samples with all other samples in the data set.** Cells with borders indicate the correct match.
(XLSX)
