# Supplementary figures and images for: Automated Genotyping of Biobank Samples by Multiplex Amplification of Insertion/Deletion Polymorphisms
Source: PLoS One. 2012 Dec 27;7(12):e52750. doi: 10.1371/journal.pone.0052750 (PMC3531329; doi:10.1371/journal.pone.0052750)

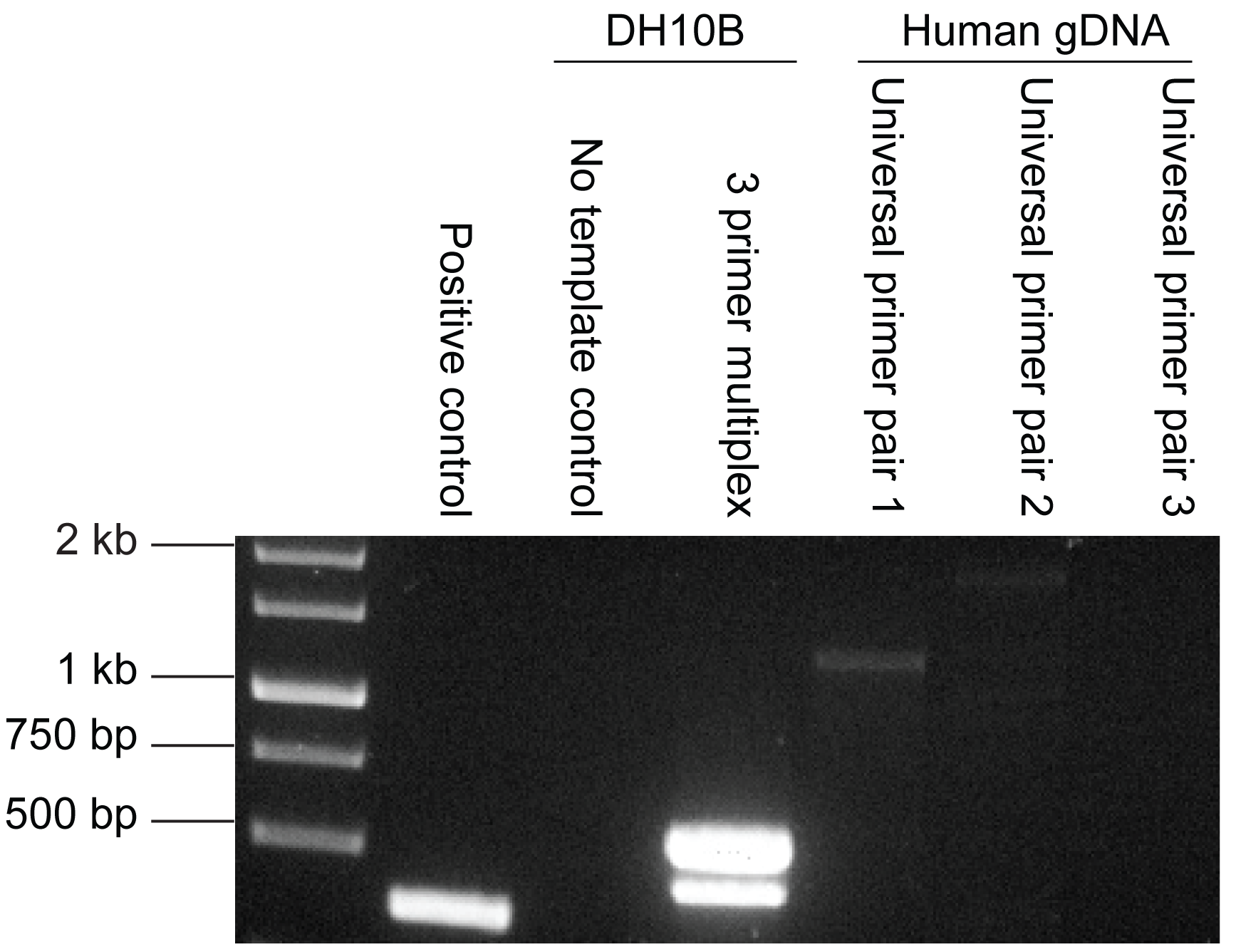

Supplement: Figure S1 — Universal primers designed against E.Coli genome do not amplify products of the same size in human gDNA. PCR products amplified using the universal primer sequences were run on a 1% agarose gel and stained with SYBRsafe. Positive control was human gDNA with amplification of PRPS1 exon 4. DH10B E.Coli DNA was used as template for the multiplex amplification of 3 primer pairs to ensure equal efficiency of primers. Human gDNA was used as template to check for unwanted PCR products of the same size as target fragments. (TIF) [file pone.0052750.s001.tif]

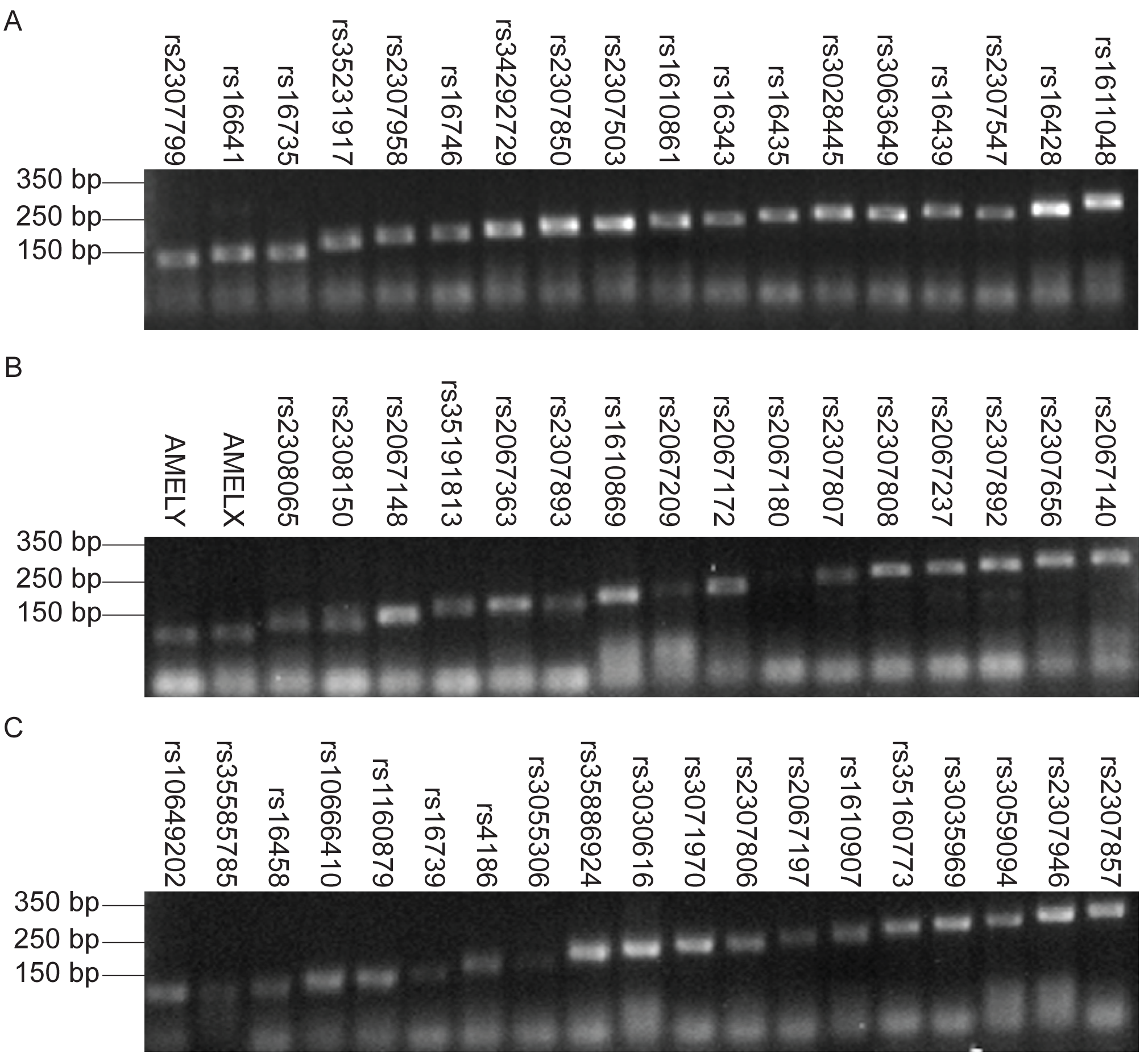

Supplement: Figure S2 — Each fragment containing a targeted insertion/deletion successfully amplified in simplex. 55 targets were amplified by a simplex MLGA reaction to ensure all could produce a PCR product before the probes were pooled. Each product was run on a 1% agarose gel. A, B and C show simplex products from panels 1, 2 and 3, respectively. (TIF) [file pone.0052750.s002.tif]

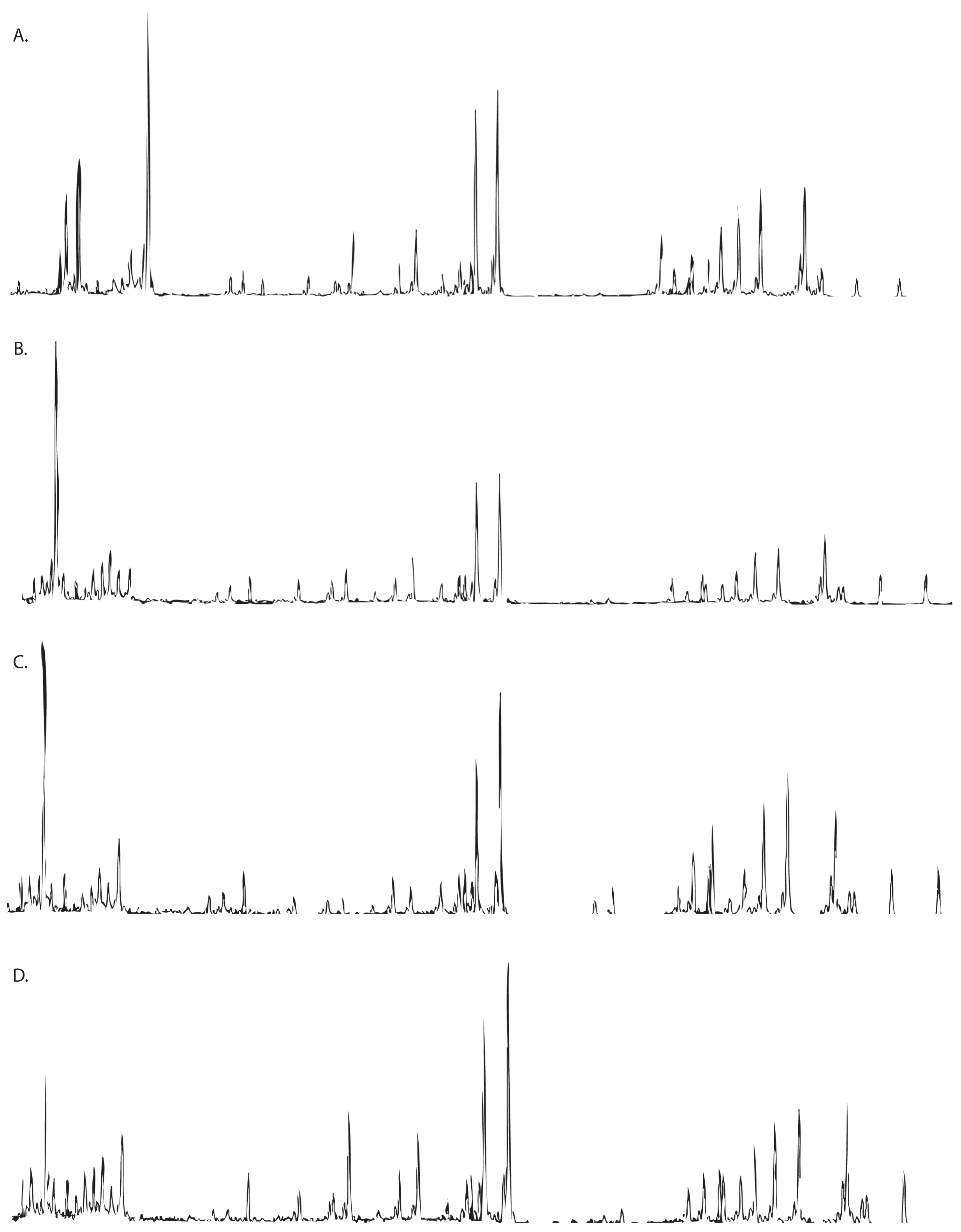

Supplement: Figure S3 — Peak profiles from SeQuanter show that the MLGA method is robust with input DNA of less than 1 ng. A, B C and D are profiles of amplified targets using input of 40, 10. 2.5 and 0,625 ng of gDNA, respectively. (TIF) [file pone.0052750.s003.tif]
