# Supplementary material for: Automated Genotyping of Biobank Samples by Multiplex Amplification of Insertion/Deletion Polymorphisms
Source: PLoS One. 2012 Dec 27;7(12):e52750. doi: 10.1371/journal.pone.0052750 (PMC3531329; doi:10.1371/journal.pone.0052750)
Supplement: Table S1 — Targeted deletions and insertions. Chromosome position according to dbSNP GRCh37, July 2011. (DOCX) [file pone.0052750.s004.docx]

| **Deletion Panel 1** | | | | | | | |
| --- | --- | --- | --- | --- | --- | --- | --- |
| **#rsNumber**  **dbSNP** | **Chromosome** | **Start** | **End** | **Deletion/Insertion** | **Product size** | **European Population**  **Prevalence/+Del** | **No. of chromosomes in prevalence study** |
| rs2307799 | NC_000005.9 | 70828426 | 70828431 | -/TTGT | 106 | 0.36 | 200 |
| rs16641 | NC_000006.11 | 141156472 | 141156476 | -/GTT | 116 | 0.56 | 200 |
| rs16735 | NC_000018.9 | 6915542 | 6915547 | -/TAGT | 123 | 0.37 | 200 |
| rs35231917 | NC_000009.11 | 25733599 | 25733604 | -/GTTT | 143 | 0.38 | 200 |
| rs2307958 | NC_000006.11 | 86035289 | 86035293 | -/CAC | 156 | 0.57 | 200 |
| rs16746 | NC_000019.9 | 39916113 | 39916117 | -/TCC | 165 | 0.37 | 200 |
| rs34292729 | NC_000001.10 | 108739438 | 108739442 | -/AGA | 178 | 0.38 | 200 |
| rs2307850 | NC_000009.11 | 135380185 | 135380190 | -/GGTG | 188 | 0.38 | 200 |
| rs2307503 | NC_000015.9 | 79826756 | 79826760 | -/TCA | 195 | 0.31 | 200 |
| rs1610861 | NC_000005.9 | 61763291 | 61763295 | -/TTA | 203 | 0.54 | 200 |
| rs16343 | NC_000004.11 | 17635559 | 17635565 | -/TTTAT | 212 | 0.62 | 200 |
| rs16435 | NC_000020.10 | 23355455 | 23355459 | -/ACT | 229 | 0.49 | 200 |
| rs3028445 | NC_000010.10 | 71197724 | 71197729 | -/TCAA | 237 | 0.35 | 200 |
| rs3063649 | NC_000010.10 | 255687 | 255691 | -/TCT | 250 | 0.63 | 200 |
| rs16439 | NC_000001.10 | 201975169 | 201975174 | -/CAGA | 259 | 0.35 | 200 |
| rs2307547 | NC_000001.10 | 7912562 | 7912567 | -/ATTT | 265 | 0.57 | 200 |
| rs16428 | NC_000018.9 | 211909 | 211914 | -/AGAG | 271 | 0.32 | 200 |
| rs1611048 | NC_000007.13 | 110939986 | 110939991 | -/TAAG | 295 | 0.43 | 200 |
| **Deletion Panel 2** | | | | | | | |
| AMELY | Y |  |  |  | 106 | N/A | N/A |
| AMELY | X |  |  |  | 109 | N/A | N/A |
| rs2308065 | NC_000007.13 | 127234354 | 127234358 | -/AGC | 119 | 0.44 | 200 |
| rs2308150 | NC_000014.8 | 73755862 | 73755867 | -/TCCT | 125 | 0.39 | 200 |
| rs2067148 | NC_000007.13 | 70067163 | 70067167 | -/ATG | 148 | 0.50 | 200 |
| rs35191813 | NC_000001.10 | 196738973 | 196738978 | -/AACA | 165 | 0.56 | 200 |
| rs2067363 | NC_000003.11 | 10316070 | 10316075 | -/AAAG | 176 | 0.39 | 200 |
| rs2307893 | NC_000018.9 | 62967665 | 62967670 | -/TAGT | 183 | 0.42 | 200 |
| rs1610869 | NC_000005.9 | 15258923 | 15258928 | -/ATTT | 191 | 0.40 | 200 |
| rs2067209 | NC_000016.9 | 84581714 | 84581719 | -/TGAA | 199 | 0.44 | 200 |
| rs2067172 | NC_000007.13 | 108202728 | 108202734 | -/ATTAA | 209 | 0.40 | 200 |
| rs2067180 | NC_000003.11 | 41130662 | 41130667 | -/TGTC | 228 | 0.53 | 200 |
| rs2307807 | NC_000013.10 | 77083473 | 77083478 | -/AACA | 234 | 0.51 | 200 |
| rs2307808 | NC_000003.11 | 97666189 | 97666193 | -/CTC | 246 | 0.32 | 200 |
| rs2067237 | NC_000012.11 | 31148722 | 31148727 | -/TGTT | 254 | 0.53 | 200 |
| rs2307892 | NC_000001.10 | 210205267 | 210205271 | -/CCT | 262 | 0.47 | 200 |
| rs2307656 | NC_000005.9 | 34844424 | 34844430 | -/TAAGT | 271 | 0.56 | 200 |
| rs2067140 | NC_000005.9 | 115887783 | 115887788 | -/CAGT | 284 | 0.61 | 200 |
| **Insertion Panel 3** | | | | | | | |
| rs10649202 | NC_000020.10 | 24401340 | 24401341 | -/GCC | 101 | 0.55 | 200 |
| rs35585785 | NC_000008.10 | 105262852 | 105262853 | -/AGTA | 107 | 0.57 | 200 |
| rs16458 | NC_000007.13 | 122151327 | 122151328 | -/TTCC | 114 | 0.69 | 200 |
| rs10666410 | NC_000008.10 | 61190688 | 61190689 | -/AGTG | 125 | 0.49 | 200 |
| rs1160879 | NC_000004.11 | 61958135 | 61958136 | -/AGA | 131 | 0.51 | 200 |
| rs16739 | NC_000017.10 | 3564696 | 3564697 | -/GAC | 139 | 0.37 | 200 |
| rs4186 | NC_000011.9 | 12552279 | 12552280 | -/TAAG | 164 | 0.32 | 200 |
| rs3055306 | NC_000001.10 | 5629608 | 5629609 | -/TCAA | 180 | 0.52 | 200 |
| rs35886924 | NC_000008.10 | 25229433 | 25229434 | -/CTAAT | 190 | 0.49 | 200 |
| rs3030616 | NC_000015.9 | 92708200 | 92708201 | -/CAAT | 203 | 0.68 | 200 |
| rs3071970 | NC_000007.13 | 50546515 | 50546516 | -/ACAAA | 210 | 0.30 | 200 |
| rs2307806 | NC_000012.11 | 67753216 | 67753217 | -/TCA | 217 | 0.38 | 200 |
| rs2067197 | NC_000005.9 | 16797105 | 16797106 | -/TTG | 234 | 0.49 | 200 |
| rs1610907 | NC_000007.13 | 110559277 | 110559278 | -/AAAGT | 240 | 0.60 | 200 |
| rs35160773 | NC_000021.8 | 24410568 | 24410569 | -/CTTG | 256 | 0.47 | 200 |
| rs3035969 | NC_000020.10 | 39152458 | 39152459 | -/ATC | 263 | 0.66 | 200 |
| rs3059094 | NC_000014.8 | 27783748 | 27783749 | -/CTA | 275 | 0.37 | 200 |
| rs2307946 | NC_000001.10 | 120185496 | 120185497 | -/GATT | 291 | 0.30 | 200 |
| rs2307857 | NC_000005.9 | 37038518 | 37038519 | -/ACAT | 299 | 0.56 | 200 |
