# Supplementary material for: Automated Genotyping of Biobank Samples by Multiplex Amplification of Insertion/Deletion Polymorphisms
Source: PLoS One. 2012 Dec 27;7(12):e52750. doi: 10.1371/journal.pone.0052750 (PMC3531329; doi:10.1371/journal.pone.0052750)
Supplement: Table S2 — Probe sequences. (DOCX) [file pone.0052750.s005.docx]

| **#rsNumber** | **Sequence Name** | **Sequence** |
| --- | --- | --- |
| rs2307799 | PROBE\|NC_000005.9_TARGET_29\|NlaIII/DdeI\|+\|4 | TTTTGCTTTCACTAGTTTTTACTACTCATGCTCGATGCGATCGAAGCTTCGGCCGATGCCAAATGTATAAATTTAATGCAACTGAATTTAAA |
| rs16641 | PROBE\|NC_000006.11_TARGET_35\|NlaIII/DdeI\|+\|4 | TTTTTTCTCTCACAAAAATGATGAGGCCTGCTCGATGCGATCGAAGCTTCGGCCGATGCCAAATCATGTAGTGTGAGTTTCCATACAGCAAA |
| rs16735 | PROBE\|NC_000018.9_TARGET_2\|NlaIII/DdeI\|+\|3 | TTTTGCTTGAAAACAAACATTTTAATCCTGCTCGATGCGATCGAAGCTTCGGCCGATGCCAAATCATGATTTGTCAAATTAGTTTTAGAAAA |
| rs35231917 | PROBE\|NC_000009.11_TARGET_6\|NlaIII/DdeI\|+\|7 | TTTAGCCTCTAATAAATATTCCTGAAGTTGCTCGATGCGATCGAAGCTTCGGCCGATGCCAAATGTAAAAGAAGTTATATTTCAAGGTCAAA |
| rs2307958 | PROBE\|NC_000006.11_TARGET_23\|NlaIII/DdeI\|+\|6 | TTTTGGAGCCTCGAGAGCAGCTCTGCTCGATGCGATCGAAGCTTCGGCCGATGCCAAATCATGGAGGACCCGGTATGAGGAAA |
| rs16746 | PROBE\|NC_000019.9_TARGET_10\|NlaIII/DdeI\|+\|4 | TTTTGGACTTCCAGGTCAAGTTCTGATGCTCGATGCGATCGAAGCTTCGGCCGATGCCAAATCATGAAAGAATCAGTATTAACAAAAAAA |
| rs34292729 | PROBE\|NC_000001.10_TARGET_16\|NlaIII/DdeI\|+\|3 | TTTTCTTCTTTCTTCCTGTGGAGCCTTTGCTCGATGCGATCGAAGCTTCGGCCGATGCCAAATGAGTAGAATGTAGTGTGAGAACAAGAAA |
| rs2307850 | PROBE\|NC_000009.11_TARGET_26\|NlaIII/DdeI\|+\|1 | TTTCACGCAGGTTGGGCATCTCATGCTCGATGCGATCGAAGCTTCGGCCGATGCCAAATCATGCTTTTAAAGTAAAGAAAGAAAAAA |
| rs2307503 | PROBE\|NC_000015.9_TARGET_2\|NlaIII/DdeI\|+\|4 | TTTAGGGATGTTGAGAGGTAAATTCTGATGCTCGATGCGATCGAAGCTTCGGCCGATGCCAAATGATGCCACTTTGCTCTGGGATTTAAA |
| rs1610861 | PROBE\|NC_000005.9_TARGET_21\|NlaIII/DdeI\|+\|1 | TTTAGGAACTATTTGGTGACTGTTCTGATGCTCGATGCGATCGAAGCTTCGGCCGATGCCAAATCATGAGGCCGCAATAATGTGAATCAAA |
| rs16343 | PROBE\|NC_000004.11_TARGET_2\|NlaIII/DdeI\|+\|3 | TTTGCCTCCTATGCAAAGAACTTTCTGATGCTCGATGCGATCGAAGCTTCGGCCGATGCCAAATCATGTAAGTGTATAAATGAAATTGTAAA |
| rs16435 | PROBE\|NC_000020.10_TARGET_4\|NlaIII/DdeI\|+\|3 | TTTTGGAGGACTTGGCAGCACAGTGCTCGATGCGATCGAAGCTTCGGCCGATGCCAAATGAGGAAAGTTTTGCTTTTTGTGGCAAAA |
| rs3028445 | PROBE\|NC_000010.10_TARGET_4\|NlaIII/DdeI\|+\|3 | TTTGCTCCAGTGATCCTGAAAGTCCTGCTCGATGCGATCGAAGCTTCGGCCGATGCCAAATCATGTGATGCATTCTTTTATTTCCTAAA |
| rs3063649 | PROBE\|NC_000010.10_TARGET_1\|NlaIII/DdeI\|+\|4 | TTTTTCAATACGCAACTCTTCCAATTATTGCTCGATGCGATCGAAGCTTCGGCCGATGCCAAATGCTGACGGGTGGTCTCTTTAGGAAA |
| rs16439 | PROBE\|NC_000001.10_TARGET_24\|NlaIII/DdeI\|+\|5 | TTTCTCTCCACTTCTTCCCAGAGACTTATGCTCGATGCGATCGAAGCTTCGGCCGATGCCAAATGGACAGGAAAATACTGTCATTCTCTAAA |
| rs2307547 | PROBE\|NC_000001.10_TARGET_4\|NlaIII/DdeI\|+\|1 | TTTTTATGTTAATTCAGTTTAAGTCTAATGCTCGATGCGATCGAAGCTTCGGCCGATGCCAAATCATGAAATTTAAAACATCATTACTTAAA |
| rs16428 | PROBE\|NC_000018.9_TARGET_1\|NlaIII/DdeI\|+\|4 | TTTGCTGGACAAATACTTGATTGTGCCTGCTCGATGCGATCGAAGCTTCGGCCGATGCCAAATGAAGACAAAAGACTTGAGGTCAAAAAAA |
| rs1611048 | PROBE\|NC_000007.13_TARGET_25\|NlaIII/DdeI\|+\|3 | TTTAACAGACCATATGCAAGTATCTTTGTGCTCGATGCGATCGAAGCTTCGGCCGATGCCAAATGAATGTCAGAGATACTTATATGTCTAAA |
| AMELY | PROBE\|NC_000024.9_TARGET_1\|NlaIII/DdeI\|+\|33 | TTTAAAACCAAATCTTCAGCTATTCTCAACGAAATCCGCGTCGAAGCTTAAACCGCACCGTTGGGTTAAACTAAAAGTTAAACAAACTAAAA |
| AMELX | PROBE\|NC_000023.10_TARGET_1\|NlaIII/DdeI\|+\|65 | TTTGAGGGACTGTTATAGCAGAAATTCAACGAAATCCGCGTCGAAGCTTAAACCGCACCGTTGGGCCAACAAAATGTCAAGAAATACCAAAA |
| rs2308065 | PROBE\|NC_000007.13_TARGET_31\|NlaIII/DdeI\|+\|5 | TTTACACACAGCTTGGCCACTCAACGAAATCCGCGTCGAAGCTTAAACCGCACCGTTGGGGCCTGGCACTGGCAGAACCAAA |
| rs2308150 | PROBE\|NC_000014.8_TARGET_14\|NlaIII/DdeI\|+\|2 | TTTGCCATAAATGAGGAGGGTTGCTTAACGAAATCCGCGTCGAAGCTTAAACCGCACCGTTGGCATGCTTTCACTTTTGCCTGTGTTAAA |
| rs2067148 | PROBE\|NC_000007.13_TARGET_10\|NlaIII/DdeI\|+\|6 | TTTAAGCTGCCTGGGAGCCCTGAACGAAATCCGCGTCGAAGCTTAAACCGCACCGTTGGGGTAGCTGTCTTGTTATCATACGTGAAA |
| rs35191813 | PROBE\|NC_000001.10_TARGET_22\|NlaIII/DdeI\|+\|2 | TTTTCGTCTCTCAACCATTTTCTTCTGAACGAAATCCGCGTCGAAGCTTAAACCGCACCGTTGGCATGTCAATCATTCTCATTACCTCTAAA |
| rs2067363 | PROBE\|NC_000003.11_TARGET_3\|NlaIII/DdeI\|+\|2 | TTTCACATCCAACCTACGACAGGTGACGAAATCCGCGTCGAAGCTTAAACCGCACCGTTGGGAGTAGTGAAATAAACTATTAGAGCAAA |
| rs2307893 | PROBE\|NC_000018.9_TARGET_11\|NlaIII/DdeI\|+\|5 | TTTGAGGCAAAACAGTATAGAGATCTGAACGAAATCCGCGTCGAAGCTTAAACCGCACCGTTGGGGTAATTTAATATTATTCAGAACAGAAA |
| rs1610869 | PROBE\|NC_000005.9_TARGET_9\|NlaIII/DdeI\|+\|9 | TTTGTTCTGGACAGATATCATAGCCTAAACGAAATCCGCGTCGAAGCTTAAACCGCACCGTTGGCATGTGACAGTGGCCAGTTTCATAAA |
| rs2067209 | PROBE\|NC_000016.9_TARGET_11\|NlaIII/DdeI\|+\|7 | TTTAGCCTCGGGAAGCAGGCTCAACGAAATCCGCGTCGAAGCTTAAACCGCACCGTTGGCATGGAGATGCCCCAGGCCTAAA |
| rs2067172 | PROBE\|NC_000007.13_TARGET_22\|NlaIII/DdeI\|+\|3 | TTTTTTTCTGAAAGTTACAGGTTCCTCAACGAAATCCGCGTCGAAGCTTAAACCGCACCGTTGGGGTGACGATGGGGGAAAAACTTAAAAA |
| rs2067180 | PROBE\|NC_000003.11_TARGET_10\|NlaIII/DdeI\|+\|4 | TTTTGTCATCTGTCTTTATGAAAGACAGACGAAATCCGCGTCGAAGCTTAAACCGCACCGTTGGCATGATGCCTTCAGACTTCACATCTAAA |
| rs2307807 | PROBE\|NC_000013.10_TARGET_7\|NlaIII/DdeI\|+\|5 | TTTGCCACTCTTCCCAGAATGCTGAACGAAATCCGCGTCGAAGCTTAAACCGCACCGTTGGGAGTATATTTGAATATGTTGAAGAAAAA |
| rs2307808 | PROBE\|NC_000003.11_TARGET_17\|NlaIII/DdeI\|+\|4 | TTTGAGGAGGAGATTTCCTGTCTTCTCAACGAAATCCGCGTCGAAGCTTAAACCGCACCGTTGGCATGTTTGGATTCACCTGTAAATTGAAA |
| rs2067237 | PROBE\|NC_000012.11_TARGET_3\|NlaIII/DdeI\|+\|2 | TTTGACGCTTGGTCACTATACCCTCAACGAAATCCGCGTCGAAGCTTAAACCGCACCGTTGGCATGTAAAGGAGGGTGGCTGCAAA |
| rs2307892 | PROBE\|NC_000001.10_TARGET_26\|NlaIII/DdeI\|+\|2 | TTTCCCCTTGCTCTTCACAAAACTCAACGAAATCCGCGTCGAAGCTTAAACCGCACCGTTGGCATGGCATTTAAATGCTCCACCAGAAA |
| rs2307656 | PROBE\|NC_000005.9_TARGET_12\|NlaIII/DdeI\|+\|2 | TTTCTCCTCCTTCGAGACACAACTCAACGAAATCCGCGTCGAAGCTTAAACCGCACCGTTGGCATGCTAATATCCTTCTAGAGTTCTAAA |
| rs2067140 | PROBE\|NC_000005.9_TARGET_53\|NlaIII/DdeI\|+\|5 | TTTGGACATTTGGTGGGTAGTGATTGGACGAAATCCGCGTCGAAGCTTAAACCGCACCGTTGGGTCTGTGTAAAGTTATGTGTTTTATAAA |
| rs10649202 | PROBE\|NC_000020.10_TARGET_5\|NlaIII/DdeI\|+\|7 | TTTGTTATCTCCATTATGGCCCAGGTGTGATGCGACGACGCAAAGCTTTCGGCGACGGTTTCACATGGAGCCAACATCCTGGCAAA |
| rs35585785 | PROBE\|NC_000008.10_TARGET_27\|NlaIII/DdeI\|+\|7 | TTTTGTGGGTATAACAGTAGTGGAATTTTGATGCGACGACGCAAAGCTTTCGGCGACGGTTTCACATGCTTGAAGCTATATTGTTTCATAAA |
| rs16458 | PROBE\|NC_000007.13_TARGET_30\|NlaIII/DdeI\|+\|10 | TTTGGTAGGTTTTTGGGGGAAGATTAACTGATGCGACGACGCAAAGCTTTCGGCGACGGTTTCAGAAGTTATAGGGCAAATATGTATACAAA |
| rs10666410 | PROBE\|NC_000008.10_TARGET_12\|NlaIII/DdeI\|+\|5 | TTTCCAACATCTGGGCCCTCTCACTGATGCGACGACGCAAAGCTTTCGGCGACGGTTTCAGATAAAATTTACTTCATCAATTTGAAAA |
| rs1160879 | PROBE\|NC_000004.11_TARGET_3\|NlaIII/DdeI\|+\|2 | TTTTCCTAATGCACATAAGTTACTCTTATGATGCGACGACGCAAAGCTTTCGGCGACGGTTTCACATGTTATGCCAATAAGATAAGTTCAAA |
| rs16739 | PROBE\|NC_000017.10_TARGET_1\|NlaIII/DdeI\|+\|5 | TTTGTCTCAAGTACAAATACTTATCTGATGATGCGACGACGCAAAGCTTTCGGCGACGGTTTCACATGTATGAGGCACGTCCTGGAAA |
| rs4186 | PROBE\|NC_000011.9_TARGET_5\|NlaIII/DdeI\|+\|4 | TTTATTTTATATGTATGTTTGTGCTTTATGATGCGACGACGCAAAGCTTTCGGCGACGGTTTCAGATTGTTATTTTAAAATGTGAAAGGAAA |
| rs3055306 | PROBE\|NC_000001.10_TARGET_1\|NlaIII/DdeI\|+\|3 | TTTTTATACTTGTGGAAAAATAAAATTATGATGCGACGACGCAAAGCTTTCGGCGACGGTTTCAGAATAAATCTAATGAAAGATATGCAAAA |
| rs35886924 | PROBE\|NC_000008.10_TARGET_8\|NlaIII/DdeI\|+\|2 | TTTACCTATGTATCCGTATTTTTTCTAATGATGCGACGACGCAAAGCTTTCGGCGACGGTTTCAGGCTAAACTTTCAGTCTAAATCCTGAAA |
| rs3030616 | PROBE\|NC_000015.9_TARGET_4\|NlaIII/DdeI\|+\|5 | TTTTTCCAGCGCCAGGAATGTGATGATGCGACGACGCAAAGCTTTCGGCGACGGTTTCAGTGCCTTCTCTAGATTTGGGCTGAAA |
| rs3071970 | PROBE\|NC_000007.13_TARGET_7\|NlaIII/DdeI\|+\|4 | TTTCAATATACAAGCTGCCCTTCCCATGATGCGACGACGCAAAGCTTTCGGCGACGGTTTCACATGTAGGCTTTGAAGATGTATTCCAAA |
| rs2307806 | PROBE\|NC_000012.11_TARGET_5\|NlaIII/DdeI\|+\|4 | TTTAGTAACAAGATATACAAGCACAATTTGATGCGACGACGCAAAGCTTTCGGCGACGGTTTCACATGCAACTCTGACACGACAGATAAA |
| rs2067197 | PROBE\|NC_000005.9_TARGET_10\|NlaIII/DdeI\|+\|3 | TTTTAAGTACTTGGGATTAGGGCTTCAGTGATGCGACGACGCAAAGCTTTCGGCGACGGTTTCAGGATGTTTGGACTTTCTGTCTAGTTAAA |
| rs1610907 | PROBE\|NC_000007.13_TARGET_24\|NlaIII/DdeI\|+\|5 | TTTAGTAAATATACTGAAAAGGGAAAGTTGATGCGACGACGCAAAGCTTTCGGCGACGGTTTCAGTTTCATTTGTGATTTCTAATATTGAAA |
| rs35160773 | PROBE\|NC_000021.8_TARGET_8\|NlaIII/DdeI\|+\|3 | TTTGTTTCATAGGTCGATTGTTTCTGTCTGATGCGACGACGCAAAGCTTTCGGCGACGGTTTCACATGTGTTTATAATGAGGCACAAAGAAA |
| rs3035969 | PROBE\|NC_000020.10_TARGET_11\|NlaIII/DdeI\|+\|5 | TTTGGGCCTGGGAAGTTGGATTCTAATGATGCGACGACGCAAAGCTTTCGGCGACGGTTTCAGGGGGATAAGCAACTGTGTCTGAAA |
| rs3059094 | PROBE\|NC_000014.8_TARGET_2\|NlaIII/DdeI\|+\|2 | TTTATGGCAGAAGAGAAGCAGTTAAACATGATGCGACGACGCAAAGCTTTCGGCGACGGTTTCACATGCTCACTATGTATTCACAGCAGAAA |
| rs2307946 | PROBE\|NC_000001.10_TARGET_17\|NlaIII/DdeI\|+\|1 | TTTAGAGAATCTAGAATAATGGGTAGTGTGATGCGACGACGCAAAGCTTTCGGCGACGGTTTCAGCACATCTTTGATGCTTGATGAAGTAAA |
| rs2307857 | PROBE\|NC_000005.9_TARGET_14\|NlaIII/DdeI\|+\|3 | TTTATACTCAAGATAAAGAACAAGAGGCTGATGCGACGACGCAAAGCTTTCGGCGACGGTTTCACATGAGCTGGGGTCTTATTTTGCTAAA |
