# Supplementary material for: Automated Genotyping of Biobank Samples by Multiplex Amplification of Insertion/Deletion Polymorphisms
Source: PLoS One. 2012 Dec 27;7(12):e52750. doi: 10.1371/journal.pone.0052750 (PMC3531329; doi:10.1371/journal.pone.0052750)
Supplement: Table S3 — Concordances of above 95% using DNA from the same non-tumor tissue. (DOCX) [file pone.0052750.s006.docx]

| **Sample** | **Concordance of duplicates (%)** | **No. of peaks compared** |
| --- | --- | --- |
| 26 | 97.9 | 94 |
| 178 | 97.9 | 94 |
| 32 | 95.9 | 98 |
| 128 | 96.9 | 96 |
| 36 | 99.0 | 98 |
| 140 | 99.0 | 92 |
| 38 | 99.0 | 98 |
| 152 | 99.0 | 92 |
